# Cone-beam computed tomography evaluation of root proximity of miniscrew implant and its correlation with failure

**DOI:** 10.1186/s12903-025-06557-3

**Published:** 2025-07-26

**Authors:** Hyeon-Shik Hwang, Hyun-Jung Lee, Seung-Weon Lim, Jin-Hyoung Cho, Till E. Bechtold

**Affiliations:** 1 Department of Orthodontics, School of Dentistry, Chonnam National University, Gwangju, Korea; 2 Arthur A. Dugoni School of Dentistry, University of the Pacific, San Francisco, CA USA; 3 Division of Orthodontics, Department of Dentistry, Hanyang University Hospital, Seoul, Korea; 4 Department of Prosthodontics, Geriatric Dentistry and Craniomandibular Disorders / Section of CMD, Center of Dentistry and Craniofacial Sciences, Charité– Universitätsmedizin Berlin, corporate member of Freie Universität Berlin and Humboldt-Universität zu Berlin, Berlin, Germany

**Keywords:** Orthodontic miniscrew implant, Root proximity, CBCT, Miniscrew failure, 3D evaluation

## Abstract

**Background:**

Orthodontic miniscrew anchorage induced a paradigm shift in orthodontics, by avoiding unwanted movement of anchoring teeth. Failure of miniscrew anchorage (premature loosening of the miniscrew) is known to be related to root proximity after insertion.

**Objective:**

This study aimed to evaluate root proximity of orthodontic miniscrew implants using CBCT and to more precisely investigate the correlation between root proximity and miniscrew failure.

**Materials and methods:**

CBCT images of 47 miniscrews, placed buccally between maxillary first molars and second premolars, were used in this retrospective study. In the reoriented CBCT images, presence and amount of root proximity were measured. Miniscrew failure was defined as loosening during the first month after placement. Chi-square test was used to evaluate the correlation between presence of root proximity and miniscrew failure rate. Logistic regression analysis was used to evaluate the statistical significance of correlation between the presence of miniscrew failure and the amount of root proximity area.

**Results:**

Among the miniscrews that showed root proximity, 52.4% failed within one month after placement, whereas the failure rate of miniscrews without root proximity was 0. According to chi-square test, miniscrew failure was 2.1 times more probable with root proximity than without. Logistic regression analysis showed statistical significance between amount of root proximity and miniscrew failure.

**Conclusions:**

This study could confirm root proximity as a major influencing factor for interradicular miniscrew failure, and present a novel approach for measuring the amount of root proximity in CBCT for preciser prediction of failure, as not every miniscrew in root proximity will fail.

## Introduction

Miniscrew implant is one of the most-widely used temporary anchorage devices (TADs) in orthodontic treatment, since it does not require complex surgical skills [1]. The clinical value of miniscrews has been proven for various insertion sites and purposes [2–5], material and design are well-engineered [6–8], and treatment results have been shown to be stable [9, 10].

Miniscrews, in general, show relatively encouraging success rates; a recent systematic review demonstrated success rates of 89.87% in the maxilla and 79.24% in the mandible [11]. However, the success rate of miniscrews is still relatively low compared with dental implants. The possibility of miniscrew failure needs to be considered in any related treatment planning [12].

Numerous clinical studies have investigated possible causes of miniscrew failure and found: lesser distance to alveolar crestal bone [13], young age of the patient [14], low level of experience of the clinician [15], laterality [16], and root proximity [17]. Kuroda et al. [17] conducted a study to evaluate root proximity using 2D radiographs; they reported a significant correlation between success rate and root proximity. Chen et al. [18] showed that miniscrews contacting the root were at great risk of failure in an animal study. Oh et al. [19] also reported that miniscrew stability decreased when the screw contacts the adjacent tooth root in beagle dogs. However, Kim et al. [20] claimed that root proximity was not a major risk factor for miniscrew implant failure in their 3D cone-beam computed tomography (CBCT) study. They reported only 1 failure in 15 mini-implants with root proximity and 1 failure in 35 without root proximity.

Motoyoshi et al. [21] also conducted a CBCT study to evaluate root proximity, and showed that more failures were seen in root contact cases; seven out of 37 miniscrews which showed root contact presented a failure. Watanabe et al. [22] performed a study to evaluate correlations between miniscrew failure rate and root proximity, insertion angle, bone contact length, and bone density using CBCT. Here, root proximity, among many variables, was most correlated with miniscrew failure, but there was no significant correlation in the maxilla. Statistically significant correlations between root proximity and miniscrew failure were present only in the mandible.

Although all these studies investigated a relationship of root proximity with miniscrew failure, the measurement was merely a distance between the screw and the adjacent tooth root [22], or a simple dichotomous measurement, that is root proximity or not [20, 21]. The amount of root proximity area was not evaluated in the previous studies. There has been no attempt to evaluate the relationship between the amount of proximity and the presence of miniscrew failure.

The purpose of this study was to evaluate root proximity of miniscrew implants using CBCT images and to further investigate the relationship between root proximity and miniscrew failure. In particular, the amount of (length along the thread being in) root proximity was measured and used in this evaluation.

## Materials and methods

This retrospective chart review was approved by the Chonnam National University Institutional Review Board. A retrospective chart review of patients was conducted consecutively at the Graduate Orthodontic Clinic of Chonnam National University Dental Hospital. The CBCT images taken immediately after miniscrew placement were used as the material in this study.

From a chart review from 2013 to 2016, those patients who had received miniscrews between the maxillary first molar and second premolar were sought out. Four hundred and twenty two miniscrews in 212 patients were selected first. Among these, 47 miniscrews in 24 patients which had CBCT images taken after their miniscrew placement were used in this study.

In these patients, a CBCT scan had been obtained, if root damage or root proximity had been suspected after miniscrew placement. If root damage had been seen in section views of a CBCT image, the screw had been removed. The miniscrews which did not show root damage had been left and used as anchorage in the orthodontic treatment. These screws with CBCT images were used as the material in this study.

The miniscrews used in this study were tapered type screws with a 1.8 mm diameter and 8 mm length (Orlus #18108, Ortholution Inc., Seoul, Korea). This type of miniscrew tapers from a larger head diameter to a smaller diameter at its tip; the screw diameter is measured at the midpoint from the neck to the tip of the screw. The material of this miniscrew implant is commonly used grade 23 titanium.

The miniscrews had been placed– with a hand-driven driver without pre-drilling– directly into the attached gingiva near the mucogingival junction between the maxillary first molar and second premolar. When inserting a miniscrew, we had given vertical angulation at 20 to 40 degrees to avoid root damage or root proximity. Horizontal angulation had been 90 degree to bone surface. For the beginning stage of screw driving, we had applied an axial force using the palm of the driving hand. After screw engagement into the bone, we had just rotated the screw without applying axial force.

Load had been applied to the miniscrews using an elastic chain on the day of miniscrew placement. Failure of miniscrew implant in this study was defined as the loosening of the screw during the first month after placement.

The CBCT scans had been obtained using a CBCT scanner (Alphard Vega, Asahi Roentgen Co., Kyoto, Japan) under the condition of 80 kV, 5 mA, voxel size 0.2 × 0.2 × 0.2 mm, and FOV 102 × 102 mm. The Digital Imaging and Communication in Medicine (DICOM) files obtained from CBCT were reconstructed into 3D images using the 3D image program (InVivoDental, Anatomage, San Jose, CA, USA).

To identify root proximity in CBCT images, section views were used. Root proximity was defined as invasion of the miniscrew into a periodontal ligament space. In this study, the determination of presence or absence of root proximity was identified through the change of section levels.

**Fig. 1:**
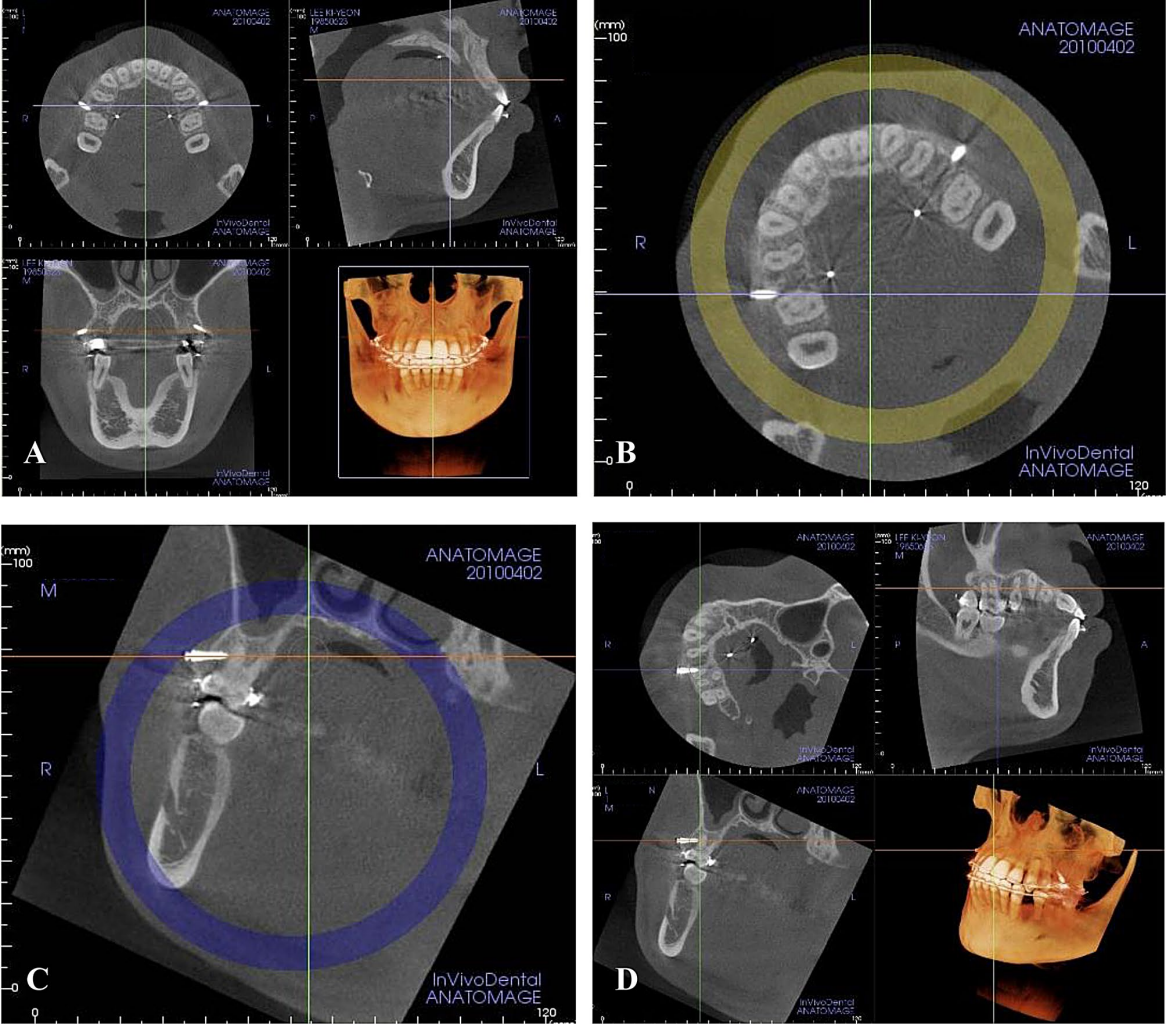
Reorientation procedure: **A**, Main window of 3D MPR view showing the axial, sagittal and frontal planes with 3D image; **B**, Adjustment of the axis of 3D image on the axial plane; **C**, Adjustment of the axis of 3D image on the frontal plane; **D**, Main window of 3D MPR view after the reorientation procedure

**Fig. 2:**
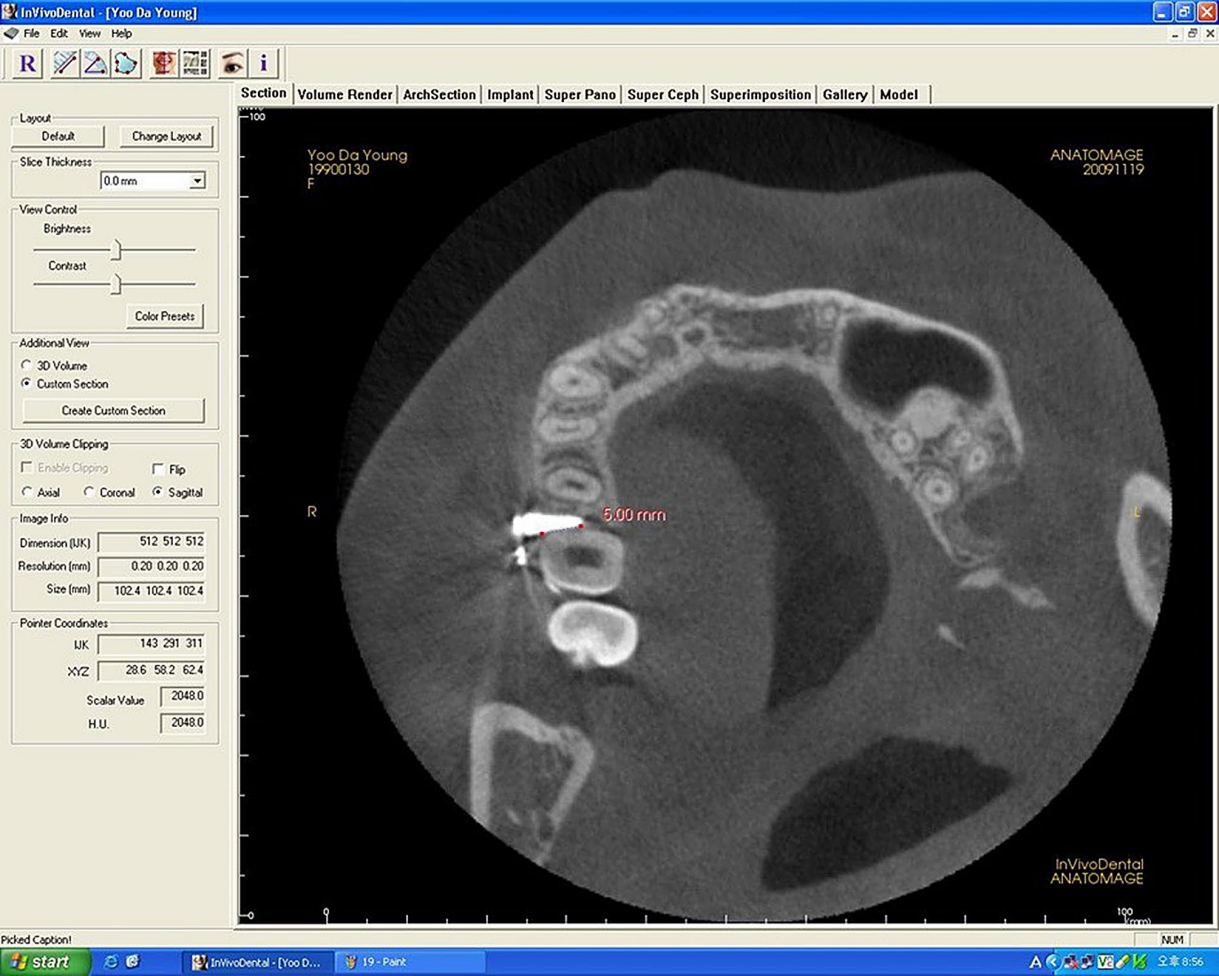
Measurement of amount of root proximity area. The screw portion which showed root proximity was measured in mm after adjusting the reference axis on MPR view identical to screw axis using reorientation in the three-dimensional image program

For the evaluation of the amount of root proximity area, all images were reoriented. The reorientation was performed by adjusting the reference axis on MPR view identical to the screw´s axis to view the total length of the miniscrew. After the reorientation procedure, the screw portion which showed root proximity was measured in mm (Figs. 1 and 2).

### Statistical analysis

In order to assess the method errors (MEs) for the measurement of the amount of root proximity, the images of 10 miniscrews were selected randomly and were reoriented. Then each root proximity area was measured twice at an interval of 2 weeks by a single investigator. The MEs were calculated using Dahlberg’s formula as follows:

$${\rm{ME = }}\sqrt {\sum {{d^2}/2n} } $$

where d is the difference between the 2 measurements and n is the number of miniscrews. The method errors in this study were 0.27 mm.

All miniscrews were classified into two groups according to the presence of root proximity. Failure rates were calculated in each group. Chi-square test was used to evaluate correlation between the presence or absence of root proximity and miniscrew failure rate.

The miniscrews which showed root proximity were divided into 2 subgroups, according to success or failure. The amount of root proximity area was computed in each group and compared between the two groups using Student *t*-test. Logistic regression analysis was used to evaluate the statistical significance between the amount of proximity and the presence of miniscrew failure. All analyses were conducted using SPSS software (version 21.0; SPSS Inc., Chicago, IL).

## Results

Twenty one of 47 miniscrews showed root proximity. Among these, 52.4% showed miniscrew failure. Failure rate was 0 in the miniscrews which did not show root proximity. Chi-square test showed that the probability of miniscrew failure was significantly higher (*p* < 0.001) in miniscrews with root proximity than in miniscrews without root proximity. Odds ratio was 2.10 indicating that the probability of failure is 2.1 times higher in miniscrews with root proximity than in miniscrews without root proximity (Table 1).

**Table 1:** Failure rate of Miniscrew placement according to root proximity

Root proximity		Insertion (*n*)		Failure (*n*)		Failure rate (%)
Present		21		11		52.4
Absent		26		0		0.0

Chi-square statistic = 15.727; *p* < 0.001: Odds ratio = 2.1

**Fig. 3:**
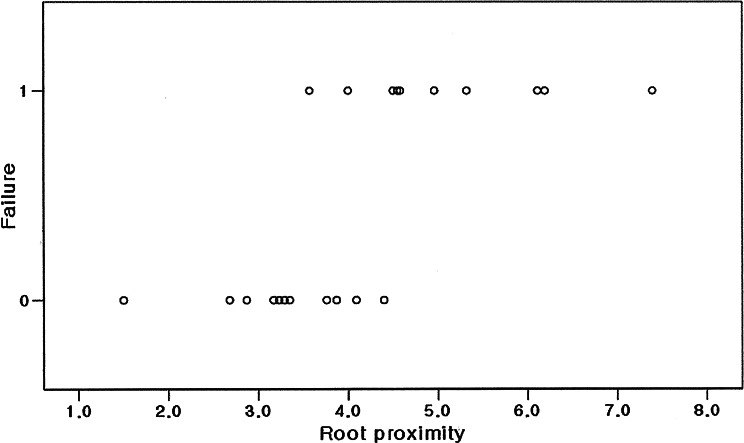
Scatter plot for miniscrew failure according to the amount of root proximity area. Logistic regression analysis showed statistical significance (*p* = 0.029) between the amount of root proximity and the presence of failure

The average amount of root proximity area was 3.54 mm in the miniscrew success group (*n* = 10), whereas the average amount of root proximity was 5.12 mm in the miniscrew failure group (*n* = 11). The difference between the two groups was statistically significant (*p* = 0.001). Logistic regression analysis showed statistical significance (*p* = 0.029) between the amount of root proximity and miniscrew failure, indicating that the probability of miniscrew failure becomes high in case the amount of root proximity area increases (Table 2; Fig. 3).

**Table 2:** Root proximity group: amount of root proximity area according to initial stability

Root proximity (n = 21)		Success		Failure		Significance (P-value)
		Mean ± SD		Mean ± SD		
Amount of root proximity (mm)		3.54 ± 0.56		5.12 ± 1.15		0.001

SD, Standard deviation

## Discussion

With this study, we were able to (i) confirm root proximity as a major influencing factor for orthodontic miniscrew failure (after interradicular insertion), and (ii) present a novel approach for measuring the amount of root proximity in CBCT images to more precisely predict miniscrew failure, as not every miniscrew in root proximity will fail.

Apart from miniscrew failure, Lim et al. [15] found the operator´s experience to be a significant influencing factor on initial stability failure of miniscrew implants. Four hundred and seven miniscrews inserted in 168 patients were analyzed. Potential confounding variables regarding the patient (gender, age, jaw, insertion site, tissue type) and the miniscrew itself (length and diameter of the miniscrews) were also examined using generalized estimating equation methods. None of these factors showed significant findings. Only the operator’s experience had a positive statistically significant effect on the success rate. The screws inserted by more experienced clinicians (more than 20 miniscrews) were found to have a higher success rate, compared with those inserted by less experienced clinicians (less than 20 miniscrews). In their retrospective study, Lim et al. [15] showed an overall success rate of 93.1%, while more experienced clinicians were found to have a 3.6-fold higher success rate during the initial healing period, than less experienced clinicians (97.5% of success rate for experienced vs. 90.4% for less experienced clinicians).

A plausible explanation seemed that experienced clinicians are better able to insert a screw with an adequate angle and therefore to reduce the possibility of root proximity. To avoid root contact or proximity, the vertical angle of insertion was recommended to be an oblique 20–40 degrees, after inserting the tip of the miniscrew perpendicular to the bone surface. In order to more exactly define ideal insertion angles, the present study was conducted to find more detailed features of root proximity leading to miniscrew failure.

In the present study, the failure of miniscrew implant was evaluated during the first month after its placement. Screws survive for a long time once they are initially stable [23]. If a miniscrew loosens after this initial period, its loosening is mostly related to other factors, such as periodontitis and oral hygiene [24], oral microbiome [25] or smoking [26]. To exclude possible confounding factors, loosening was evaluated only during the first month after placement.

Among the screws which showed root proximity, 52.4% showed failure. On the other hand, none of the screws which did not show root proximity loosened. These results suggest that root proximity is a major risk factor for miniscrew failure. Motoyoshi et al. [21] and Watanabe et al. [22] also showed that root proximity is highly correlated with miniscrew failure in their CBCT studies. However, Kim et al. [20] claimed that root proximity was not a major risk factor for miniscrew failure in their CBCT study. The lack of association of root proximity with miniscrew failure seems to be partly due to a small number of failures in their study. They presented 1 failure in 15 mini-implants with root proximity and also 1 failure in 35 without root proximity. It is noted that they used osseointegration-based mini-implants in their study. Considering that, most miniscrew implants on the market are not osseointegration-based, the claim of their study about little association of root proximity with miniscrew implant cannot be generalized.

Literature review reveals that miniscrew success rate is lower in the mandible than in the maxilla [11, 17]. A lower success rate in the mandible can be explained with an increased probability of root proximity in the mandible. While vertical angulation can more easily be given in the maxilla to avoid root proximity, vertical angulation in the mandible is likely to lead to slippage when placing a screw: The mandibular alveolar bone is more dense and has a steeper slope. In consequence, there is an increased probability of root proximity in the mandible. This is particularly true in cases where the attached gingival zone is narrow.

In the present study, logistic regression analysis showed statistical significance between the degree of root proximity and miniscrew failure: the amount of root proximity was measured along the length of the thread, showing an average of 3.54 mm of root proximity in the success group versus 5.12 mm in the failure group. The difference between the two groups was statistically significant, confirming that not only the incidence of root proximity itself, but a certain amount of root proximity was highly correlated with miniscrew failure.

The results of the present study confirm that root proximity should be avoided when placing miniscrews. At an early stage of miniscrew practice, a stent was suggested in order to place a screw in the accurate position and with an appropriate angulation [27, 28]. However, a CBCT scan is needed to fabricate such a stent, and a routine scan is to be seen critically in terms of radiation hygiene.

Therefore, we suggest an alternative approach: A screw could first be placed without a CBCT scan. If root damage or root proximity is suspected after the placement, a CBCT scan can be performed to check whether root damage or proximity is present. If root damage is found or the amount of root proximity is large, the screw is removed and can be placed at a different site or in a different angulation to avoid root proximity. This way, CBCT evaluation is not performed in all cases, but only in cases of suspected root damage. The frequency of CBCT scans will decrease as the clinicians develop their experience [15].

As clinical studies on miniscrews involve patients with specific needs, it is especially difficult to design randomized, prospective studies. The retrospective character of this study was idealized through detailed treatment protocols and experienced clinicians. On the other hand, the fact that only specific miniscrews were recorded on CBCT, depending on the subjective judgement of an experienced clinician, does bear a certain selection bias, to the benefit of radiation hygiene.

Within the limitations of these circumstances, the present study confirmed the correlation between root proximity and loss of stability during the first month after insertion, and was even able to introduce a novel measurement approach to define differences in the severity of root proximity that may lead to miniscrew failure or not.

Additional studies are needed to identify the causes of root proximity such as screw insertion site, vertical angle of insertion, and horizontal angle of insertion; patient-related causes could be added to create a mixed effect model. A study design with more time points could provide more detailed information on the development of too early miniscrew loss.

As an independent variable, the only variable in this study was root proximity. Additional studies with other potential causative factors could be used to design a mixed effect model. This study investigated miniscrews which were placed in the maxillary buccal molar area.

Further studies would be needed to identify if similar results can be drawn when placing a screw in other areas, such as palatal slope and mandibular buccal area.

## Conclusion

The probability of miniscrew failure is higher in miniscrews with root proximity than in miniscrews without root proximity. In addition, the probability of failure increases with the length of the thread segment which is located in root proximity.

These results indicate that root proximity is highly correlated with miniscrew failure, and the probability of failure can be predicted measuring the amount of root proximity.

## Data Availability

The datasets obtained and analyzed during the current study are available from the corresponding author upon reasonable request.
